# Cauterization as a Simple Method for Regeneration Studies in the Zebrafish Heart

**DOI:** 10.3390/jcdd7040041

**Published:** 2020-10-03

**Authors:** Papa K. Van Dyck, Natasha Hockaden, Emma C. Nelson, Alyssa R. Koch, Kamil L. Hester, Neil Pillai, Gabrielle C. Coffing, Alan R. Burns, Pascal J. Lafontant

**Affiliations:** 1 Department of Biology, DePauw University, Greencastle, IN 46135, USA; pvandyck_2020@depauw.edu (P.K.V.D.); nhockade@iu.edu (N.H.); emmanelson_2020@depauw.edu (E.C.N.); alyssakoch_2022@depauw.edu (A.R.K.); kamilhester_2020@depauw.edu (K.L.H.); neil.charles.pillai@drexel.edu (N.P.); gcoffing@uoregon.edu (G.C.C.); 2 College of Optometry, University of Houston, Houston, TX 77204, USA; arburns2@central.uh.edu; 3 Buehler Biomedical Imaging Center, DePauw University, Greencastle, IN 46135, USA

**Keywords:** cardiac, myocytes, infarction, remodeling, inflammation, collagen, nerves

## Abstract

In the last two decades, the zebrafish has emerged as an important model species for heart regeneration studies. Various approaches to model loss of cardiac myocytes and myocardial infarction in the zebrafish have been devised, and have included resection, genetic ablation, and cryoinjury. However, to date, the response of the zebrafish ventricle to cautery injury has not been reported. Here, we describe a simple and reproducible method using cautery injury via a modified nichrome inoculating needle as a probe to model myocardial infarction in the zebrafish ventricle. Using light and electron microscopy, we show that cardiac cautery injury is attended by significant inflammatory cell infiltration, accumulation of collagen in the injured area, and the reconstitution of the ventricular myocardium. Additionally, we document the ablation of cardiac nerve fibers, and report that the re-innervation of the injured zebrafish ventricle is protracted, compared to other repair processes that accompany the regeneration of the cauterized ventricle. Taken together, our study demonstrates that cautery injury is a simple and effective means for generating necrotic tissue and eliciting a remodeling and regenerative response in the zebrafish heart. This approach may serve as an important tool in the methods toolbox for regeneration studies in the zebrafish.

## 1. Introduction

Cardiovascular diseases remain the number one cause of death in the economically developed world [1]. They comprise genetic and lifestyle-linked diseases such as atherosclerosis that can lead to stroke and myocardial infarction (MI). The incidence of cardiovascular diseases is global and has been on a steep rise in many developing countries [2,3]. In the heart, occlusion of coronary arteries from the rupture of unstable atherosclerotic plaques results in oxygen deprivation that can lead to the death of myocardial tissue and significant loss of function. Effective clinical therapy can preserve remaining function or significantly delay further functional decline [4]. However, the development of heart failure remains a common sequela of MI. The available literature on mammals demonstrates that the adult heart has very limited or non-existent ability to regenerate damaged myocardial tissue [5,6]. This limitation is due in whole or in part to the terminally differentiated state of adult ventricular cardiac myocytes [7,8,9]. To date, clinical therapies that aim at recovering myocardial function following MI are limited. While approaches to myocardial tissue regeneration in humans is an area of significant research interest [10], progress is impeded by gaps in our understanding.

The heart’s response to MI modeled in the adult mouse is attended by a non-regenerative repair response. The infarcted region proceeds through sequential and temporally overlapping processes of tissue necrosis, inflammation, and granulation tissue formation, ultimately evolving into a complex and dynamic scar [11,12]. The essential contractile function of the damaged myocardium is not recovered. As the ventricle dilates, the ejection fraction steadily decreases, leading to progressive heart failure. Recent studies in neonatal mice have established the strong ability of early cardiac myocytes to re-enter the cell cycle and for the neonatal mammalian heart to regenerate prior to seven days of age [13,14]. Other studies, however, have challenged the extent of neonatal heart regeneration [15,16]. In contrast to research on adult mammals, seminal studies on amphibians have documented the ability of newts and salamanders [17,18] to regenerate damaged myocardium. Among aquatic vertebrate species, cardiac regenerative capacities have also been well documented [19]. This ability is supported by cell cycle re-entry and proliferation of cardiac myocytes. Of the regenerative species, zebrafish remain the principal animal model used in heart regeneration studies [20].

Myocardial infarction has been modeled by a variety of approaches. In mice, necrosis and loss of myocardial tissue is induced primarily through permanent coronary occlusion, occlusion and reperfusion injury, cauterization, or cryoinjury [21,22,23,24]. Myocardial stress and apoptosis can also be induced via short intermittent periods of occlusion and reperfusion [25]. While the ultimate outcome of these approaches is non-regenerative repair of the myocardium, the different injury methods resulted in significant changes in temporal expression of genes and in cardiac remodeling [26]. In seminal studies of amphibians, the regenerative response was produced via mechanical crushing or resection of the ventricular apex [27,28,29]. The first studies in adult zebrafish [19,30] involved the amputation and removal of the ventricular apex, resulting in regeneration of the missing tissue. This approach remains one of the most often used in regeneration studies since its inception more than 15 years ago. Other approaches have included ventricular perforation and scratch injury [31,32,33], and genetically programmed cell death [34,35]. Cryoinjury was shown to be effective at inducing regenerative responses in the presence of necrotic tissue and is widely used [36,37,38].

Our lab previously studied cardiac regeneration in the giant danio (*Devario. cf aequipinnatus*) and goldfish using cautery injury to produce a significant amount of necrotic myocardial tissue [39]. However, to date, the application of this method has not been performed in zebrafish. While cryoinjury is an effective means at generating volumetrically significant and localized injury, the necessary liquid nitrogen may not be readily available in small laboratories and at primarily undergraduate institutions with limited research infrastructure. However, a standard, or a refillable and portable Bunsen burner, and nichrome inoculating needles for microbiology are basic equipment that are found in most laboratories, and that can be used for thermal injury. We hypothesize that cardiac cauterization will trigger a regenerative response in the zebrafish heart. In this study, we demonstrate that this simple and effective approach to injury in the zebrafish is accompanied by necrosis, inflammation, remodeling, and regeneration. In addition, we show a role of scanning electron microscopy in the assessment of repair and regeneration. Moreover, we show that the loss of innervation at the site of injury is recovered by 120 days post-cauterization (dpc).

## 2. Materials and Methods

### 2.1. Animals

For these experiments, zebrafish aged 6 months to 1 year old, measuring an average of 28.5 mm in standard length and 482.9 mg in wet body weight, were used. Surgeries were performed in cohorts averaging seven to eight experimental fish at a time, with a survival rate ranging from 80 to 100%, for an overall average of 87.3% (*n* = 142). Adult zebrafish were raised in 3 or 10 L tanks (at a maximum of three fish per liter) in an Aquatic Habitat system (Pentair, Apopka, FL, USA) at a temperature of 28.5 °C and were kept on 14/10 h day/night light cycles. System water was maintained at a pH of 7.00 and hardness of 800 µS, with automated water exchange of 10% per day. Adult zebrafish were fed primarily Zeigler Adult Zebrafish Diet (Zeigler, Gardners, PA, USA) twice a day and supplemented twice a week with brine shrimp hatched from artemia cysts (INVE Aquaculture Inc., Salt Lake City, UT, USA) or frozen Omega Super brine shrimp (OmegaSea, Painesville, OH, USA). Experimental procedures were approved by the DePauw University Institutional Animal Care and Use Committee on 16 July 2015 (Project identification Code: ZebrafishStudyProtoc-Heart-Rev4BF). 

### 2.2. Surgery and Cautery Injury

Surgery and cauterization of the adult giant danio heart and goldfish in our lab have previously been described [39,40] and were applied to the zebrafish heart with minimal modification. Briefly, the cauterization probes used for ventricular injury were made of 25-gauge (0.455 mm) nichrome inoculating needles (Carolina Biological Supply Company, Burlington, NC, USA). The tips of the wires were polished using an 80-grit aluminum oxide Gator sandpaper sheet (Ali Industries Inc., Fairborn, OH, USA) after multiple uses to keep the tip smooth and free of debris. Additionally, 7 to 8 mm of the distal tip was bent at 90 degrees.

For surgery, adult zebrafish were first anesthetized while submerged in 0.02% buffered MS tricaine, (Sigma-Aldrich, St. Louis, MO, USA) for 30 s to a minute, then fit supine into a slit cut in a sponge block such that the ventral aspect of the fish was facing up under a Leica ZOOM 2000 dissecting microscope (Leica Microsystems, Bannockburn, IL, USA). Using a pair of Dumont #5 fine-point tweezers (Electron Microscopy Sciences, Hatfield, PA, USA), the ventral scales were removed; then the skin was perforated, and the pectoral muscles were gently dissected midline, and held open with the forceps tips to create an approximately 3 mm opening that exposed the silvery pericardium. Following the gentle dissection of the pericardial membrane, the heart ventricle and its apex were identified. During the procedure, a plastic Pasteur pipette was used to wet the sponge around the fish with system water at 30 s to 1 min intervals to prevent dehydration.

The custom nichrome wire probe was immediately introduced into the open flame of a Bunsen burner for 4 to 6 s then brought to close proximity to the heart, allowing for cooling of the probe for 4 to 5 s. The tip of the heated probe was left hovering close over the apex of the beating ventricle and was moved closer during ventricular contraction (downward motion) so that the beating ventricle made contact with the heated probe during relaxation (upward motion). Before removal of the probe from the apical ventricular surface, the probe was allowed to cool further for 4 to 5 s. Following this procedure, the fish were returned to a new tank with system water. Next, a plastic Pasteur pipette was used to aerate the water and generate water flow for 45 s to a minute over the anesthetized fish to speed up recovery from the anesthetic. For analysis, adult zebrafish were euthanized at 24 h, 3, 7, 14, 30, 60, and 120 days post-cautery (hpc, dpc) using 0.2% buffered MS tricaine (Sigma-Aldrich, St. Louis, MO, USA). Body weight and standard length of the fish were measured once all body and opercular movements had stopped. The hearts were removed by first severing the atrium at the sinus venosus–atrial junction. Then, using the forceps tips, the aorta was pinched at a segment proximal to the bulbus and gently pulled to excise the ventricle.

### 2.3. Imaging and Measurements of Injury Size by Brightfield and Scanning Electron Microscopy

Excised hearts were transferred into 1X PBS at room temperature and allowed to beat to evacuate luminal blood. Hearts were immediately imaged under brightfield microscopy using a Leica S6D dissecting microscope equipped with a Leica MC170 HD camera (Leica Microsystems, Bannockburn, IL, USA). Following imaging, the hearts were immediately fixed in 4% paraformaldehyde (PFA) in 1X PBS for further processing. Gross estimation of injury was performed by displaying the brightfield images of whole hearts on a Dell 17-inch monitor overlaid with a point-counting grid with 280 equidistant point intercepts. Injured area fraction was calculated based on the fraction of point intercepts landing randomly on the cauterized and clotted, or repairing, area to the total number of point intercepts covering the sagittal projection of the ventricular field.

For scanning electron microscopy imaging, the euthanized zebrafish were first injected ventrally, near the base of the pectoral fin, with 50 microliters of heparin sulfate 1000 USO units per mL (Sagent Pharmaceuticals, Schaumberg, IL, USA). The injected heparin was allowed to circulate for one minute. The atrium and aorta were severed and the hearts were left to beat for another minute to allow for clearing of the luminal blood. The hearts were then fixed in 4% PFA and kept at 4 °C overnight. Next the hearts were sectioned sagittally using a clean microtome knife to produce two equal halves. For dehydration, the sectioned hearts were transferred to a covered 24-well plate (Santa Cruz Biotechnology, Dallas, TX, USA) and were subjected to three exchanges of 100% ethyl alcohol in a fume hood. Next, the alcohol was substituted with increasing concentrations of hexamethyldisilizane (HMDS, Sigma-Aldrich, St. Louis, MO, USA) in ETOH, from 33.3, 66.6, and 100%, three times, for 30 min, for each concentration. Following the exchanges in 100% HMDS, the hearts were left uncovered in the 24-well plate to dry overnight. The samples were next coated with 80% palladium–20% gold using an MCM-100 ion sputter coater (SEC, Gyeonggi-do, Korea) and imaged on a NanoImages SNE-4500M (NanoImages, Pleasanton, CA, USA). The estimation of myocardial tissue recovery was performed using the point intercept method described above. Myocardial tissue area fraction was calculated based on point intercepts landing randomly on trabeculae and compact myocardium (excluding amorphous necrotic and remodeling tissues) and point intercepts covering the entire ventricle.

### 2.4. Characterization of Injury and Repair by Transmission Electron Microscopy (TEM)

For TEM, zebrafish hearts were fixed in 2.5% fresh glutaraldehyde in 100 mM cacodylate buffer overnight at 4 °C, then washed in cacodylate buffer. Next, the hearts were post-fixed in 1% tannic acid, then 1% osmium tetroxide, dehydrated in acetone, and embedded in Embed-812 resin (Electron Microscopy Sciences, Hatsfield, PA, USA). Two-micron thick sagittal sections were cut and stained with toluidine blue for light microscopy analysis. Ultrathin sections (100 nm thick) were mounted on single-slot or 200-mesh copper or nickel grids and imaged on a Tecnai G2 Spirit BioTWIN electron microscope (FEI Company, Hillsboro, Oregon).

### 2.5. Characterization of Injury and Repair by Histochemistry (Collagen and Inflammatory Cells)

Adult hearts were fixed in ice-cold 4% paraformaldehyde for 3 h at room temperature, or overnight at 4 °C. The hearts were then cryoprotected in 30% sucrose and placed in 13 mm diameter aluminum seal cups (Wheaton, Millville, NJ, USA), filled with freezing medium (Tissue-tek OCT, Torrance, CA, USA) and stored at −80 °C before sectioning. Sagittally sectioned 10 μm slices were obtained using a Leica CM 1900 cryostat (Leica Microsystems, Bannockburn, IL, USA). Cryosections from the middle of each heart containing the cauterization site were stained to detect inflammatory activity using the Peroxidase (myeloperoxidase, MPO) Leukocyte kit, according to the manufacturer’s protocol (Sigma-Aldrich, St. Louis, MO, USA). Briefly, the slides were washed gently in running tap water for 15 to 30 s and allowed to air dry. Fifty mL of Trizmal buffer was warmed to 37 °C. Peroxidase indicator reagent and 200 μL of 3% hydrogen peroxide were added to the Trizmal buffer. Slides were incubated in peroxidase indicator reagent solution at room temperature for 30 min in the dark. Slides were then washed in gently running tap water for 15 to 30 s and allowed to air dry. The MPO-stained slides were then counterstained with eosin, dehydrated in an alcohol–xylene, and coverslipped using Permount. MPO-positive cells were identified and counted at 40X magnification in the ventricular apical region in two to three fields and averaged in sections representative of each heart.

For collagen visualization, Masson’s trichrome staining and Fast Green/Sirius Red staining were carried out following the manufacturer’s protocol (Sigma-Aldrich, St. Louis, MO, USA). Briefly, slides were incubated in Bouin’s solution at room temperature overnight and stained with acid hematoxylin solution for 5 min. Next, they were stained in 0.1% Biebrich Scarlet–0.1% Acid Fuchsin in 1% acetic acid, incubated in 10% phosphotungstic–phosphomolybdic acid solution, then in 2.4% Aniline Blue solution. For Fast Green/Sirius Red staining, sections were fixed with pre-warmed Bouins’ solution overnight at room temperature, then stained with 0.1% Fast Green (Fisher Scientific, Waltham, MA, USA) for 10 min. Following a 2 min incubation in 1% acetic acid, sections were stained with 0.1% Sirius Red (Sigma-Aldrich, St. Louis, MO, USA) in saturated picric acid for 30 min. Sections were dehydrated in graded alcohol, then coverslipped using Permount mounting media (Fisher Scientific, Waltham, MA, USA). Volume density of collagen was quantitated using 280-point grid point intercepts, and was obtained by calculating the number of point intercepts randomly intersecting collagen fibers (red/orange) divided by the total number of point intercepts intersecting green-stained myocardial tissue.

### 2.6. Nerves and Fluorescence Imaging

For wholemount nerve fiber staining, adult hearts were fixed in 4% paraformaldehyde for 3 h at room temperature, or overnight at 4 °C. The hearts were rinsed in 1X PBS and permeabilized overnight in 0.5% Triton X-100 in 1X PBS. Samples were incubated for 48 to 72 h in zn-12 primary antibody (1:50, Developmental Studies Hybridoma Bank, deposited by Trevarrow, B) in 3% BSA at 4 °C. Samples were rinsed in 1X PBS before incubation in anti-mouse FITC secondary antibody (F0257, Sigma-Aldrich, St. Louis, MO, USA) for 3 h. For wheat germ agglutinin (WGA) staining, the hearts were incubated at 4 °C for 3 h in in 400 μL of lectin-containing Tris buffer saline (50 mM Tris, 150 mM NaCl, 1 mM CaCl2, 1 mM MgCl2, pH 7.6) at a final concentration of 2 μg/mL. The hearts were then washed in PBS, and nuclei were stained with Hoechst stain (Invitrogen, Eugene, OR, USA), washed three times in 1X PBS, and imaged. Adult hearts were mounted in glass-bottom tissue culture dishes (MatTek, Ashland, MA, USA), then stabilized with 2% low-melting agarose (Sigma-Aldrich, St. Louis, MO, USA). Whole hearts were imaged at 5X and the apical region at 10X using a Zeiss AxioImager M equipped with an ApoTome.2 for optical sectioning and ZEN imaging software (Zeiss, Göttingen, Germany). Image stacks were combined into three-dimensional projections and captured. Images of the apical region were scaled to fit a 280-point grid with equidistant point intercepts. Nerve fiber volume density was calculated by the number of point intercepts randomly intersecting a nerve divided by the total number of point intercepts randomly intersecting the heart.

### 2.7. Statistical Analysis

Collected data are expressed as means and standard error of means (SEM). An ordinary one-way ANOVA followed by Tukey’s post hoc test was used to analyze data on injury sizes in brightfield images and scanning electron micrographs, volume density of nerve fibers, and inflammatory cell accumulation. A *p* value of ≤0.05 was considered significant. Analysis and graphing were performed using GraphPad Prism 7 (GraphPad, La Jolla, CA, USA).

## 3. Results

### 3.1. Gross Characterization of ZebrafishHeart Subjected to Cautery Injury

**Figure 1:**
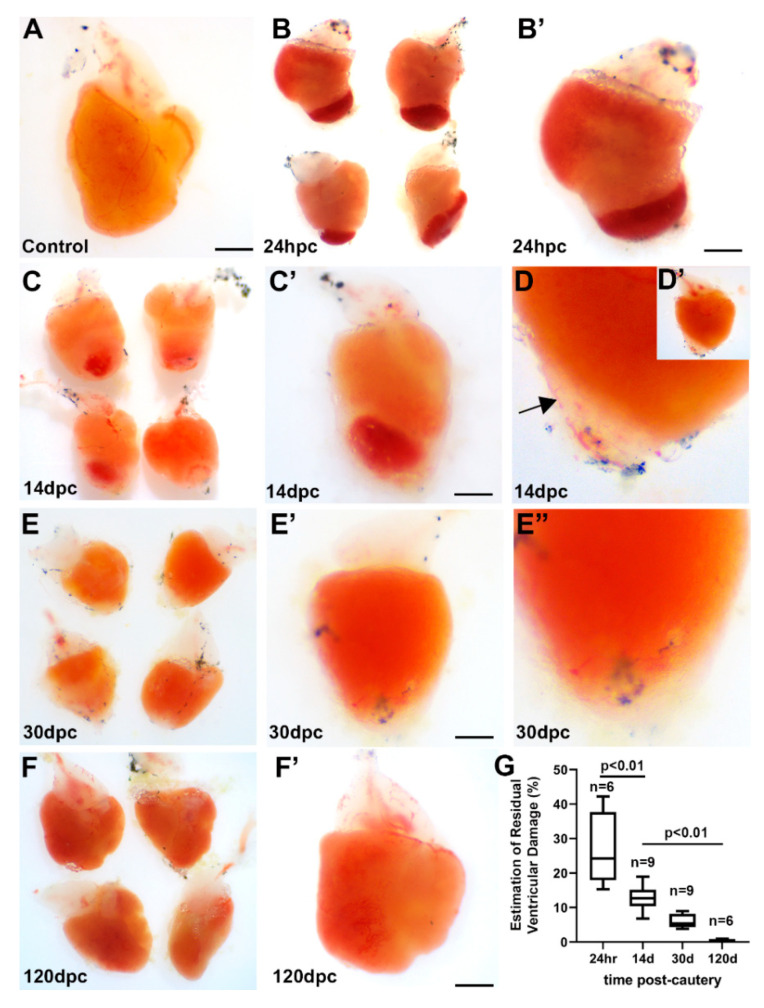
Gross characteristics of the injured and repairing zebrafish heart. Image of a non-injured excised adult zebrafish heart (**A**), and examples of four hearts 24 h post-cautery (hpc) showing a distinct area of injury (**B**). One of the hearts in panel B at higher magnification (**B′**) shows a clear demarcation between the clotted and normal-appearing tissue. Four hearts display the presence of ventricular clot at 14 days post-cautery (dpc) (**C**) as well as a region of white translucent tissue at the apical region surrounding the clot. One of the hearts at 14 dpc (**C′**) contains an achromatic area surrounding the clot. The ventricle of a 14 dpc heart at a lower and a higher magnification (**D**,**D′**) contains a blood vessel (arrow) coursing over the clear repairing area. A clot is no longer observed within hearts at 30 dpc, and the repairing area is markedly decreased (**E**,**E′**,**E″**). Injured hearts at 120 dpc (**F**) appear grossly similar to the uninjured heart in (**A**), except for occasional indentation in the apical area (**F′**). Quantitation (**G**) shows a progressive and significant decrease in the injured area. Scale bars = 500 µm.

Tools, typical setup, and approach to the performance of adult zebrafish ventricular cauterization are illustrated in Supplemental Materials, Figure S1. When subjected to cauterization, the uninjured adult zebrafish heart (Figure 1A) showed a well-defined injury (Figure 1B) that encompassed a quarter to a third of the ventricle at 24 h post-cauterization (hpc). The heat transferred from the direct application of the nichrome probe caused an immediate clot (dark red) that defined the approximate size of the injury localized at the apex or at the apicoventral aspect of the ventricle. The mid-ventricular and base region of the heart displayed a red-orange color similar to that of the uninjured ventricle (Figure 1B′). By two weeks, the area of injury was defined by the persistent presence of the clot (Figure 1C) as well as an area of blanched or achromatic, and translucent, tissue surrounding the clot (Figure 1C′). At higher magnification under brightfield, the translucent area could be seen to contain blood vessels (Figure 1D,D′). By 30 days post-cauterization (dpc), the original clot had disappeared (Figure 1E). The achromatic apical area, while decreased, could still be observed (Figure 1E′,E″). At 120 dpc, neither a clot nor a blanched area could be observed at the ventricular apex or apicoventral area (Figure 1F). The injured ventricle appeared to be grossly reconstituted. However, small areas of indentation or extracardiac tissue adhesion remained as a sequela of the original injury (Figure 1F′). By this gross measure, initial myocardial injury averaged a volume density of 26.9% soon after injury, decreasing to 14.4% by 14 dpc, 5.9% by 30 dpc, and less than 1% by 120 dpc (Figure 1G).

### 3.2. Characterization of ZebrafishCauterized Heart by SEM

**Figure 2:**
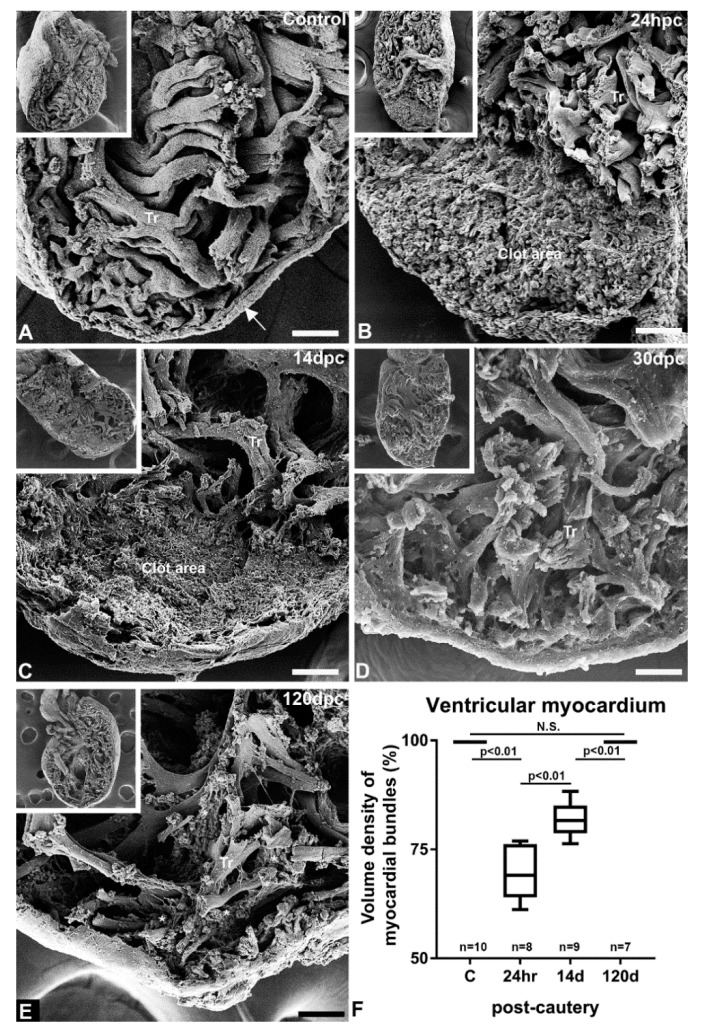
SEM characterization of the injured and regenerating heart. (**A**) Trabeculated myocardium (Tr) of the apical region bounded by a thin compact myocardium (arrow) in an uninjured heart on a sagittal section (inset). (**B**) Apical region of the ventricle with loss of trabecular structures 24 h post-cautery (hpc), whole heart (inset). (**C**) Absence of trabecular structure in the apical region at 14 days post-cautery (dpc), whole heart (inset). (**D**) Presence of trabeculae in the ventricular lumen in the apical region at 30 dpc. (**E**) Presence of trabeculae in the ventricular lumen in the apical region at 120 dpc at a higher density, with remnant of amorphous material (asterisk). Whole heart (insert). (**F**) Quantitation of compact and trabeculated myocardium in the injured and regenerating ventricle showing progressive disappearance of the necrotic area and return of apical ventricular myocardium. Scale bar = 200 µm.

To gain a better understanding of the internal structural changes that accompanied the response to cauterization, we studied the injured ventricle using scanning electron microscopy (SEM). The non-injured ventricle (Figure 2A) displayed the expected characteristics of the adult zebrafish. A thin compact heart (or cortical myocardium) enclosed the relatively thick network of trabeculae occupying the ventricular lumen (spongy heart). At 24 hpc, the base and mid-ventricular region consisted of thick bundles of trabeculae similar to the non-injured control heart. However, the apical to the lower mid-ventricular region was characterized by the absence of trabeculae, and consisted of volumes of amorphous material indicative of a fairly large clot (Figure 2B). At 14 dpc, the ventricle was characterized by the absence of observable trabeculae in the injured area. The clot was still present (Figure 2C), however, the projected texture of the original clot had changed, appearing more porous. At 30 dpc, trabecular bundles could be seen in the apical region (Figure 2D). The inner space of the apical region was grossly reconstituted with trabecular myocyte bundles. At 120 dpc, the injured and repaired area was similar to that seen 30 dpc, with small scatterings of amorphous materials and adhering red blood cells (Figure 2F). Quantitation of the myocardial muscle of the ventricle shows a volume density of 70% soon after injury, nearly 80% by 14 days, and fully regenerated by 120 dpc (Figure 2G).

### 3.3. Ultrastructural Characterization of the Injury Response by TEM

**Figure 3:**
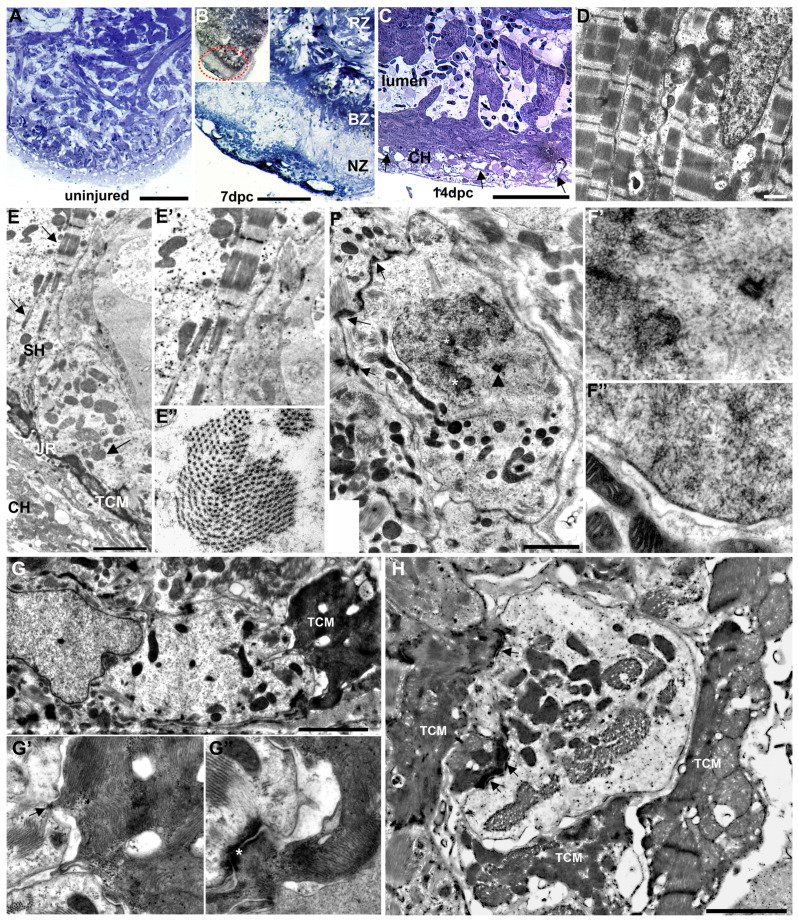
Ultrastructural characterization of the cauterized heart. (**A**) Toluidine-stained section of an uninjured zebrafish heart. (**B**) Section of a cauterized ventricle (inset) at 7 days post-cautery (dpc) showing the apical necrotic zone (NZ), the remote basal zone (RZ), and the border zone (BZ) intercalated between the two at higher magnification. The section shows a reduction in stain intensity in the cauterized apical region consistent with the loss of sarcomeric myocardial structure. (**C**) Regenerated region 14 dpc showing compact myocardium with numerous coronary vessel profiles (arrows) in the compact heart (CH) and myocardial trabeculae projecting into the ventricular lumen. (**D**) TEM of well-organized and sarcomere-rich cardiac myocytes in the basal region remote to the injury. (**E**) TEM 14 dpc of the junctional region (JR) at the interface between the compact heart (CH) and spongy heart (SH) with an electron-dense transitional cardiac myocytes (TCMs). Sparse sarcomeres (arrows) with various levels of organization can be seen in the trabecular cardiac myocytes of the spongy heart. The compact heart appears less organized. Higher magnification of longitudinally sectioned sarcomeres (**E′**), and cross-sectioned sarcomeres (**E″**) in trabecular myocytes in (**E**). (**F**) A mitotic cardiac myocyte profile with sparse and poorly organized sarcomeric structures compared to adjacent myocytes. The mitotic myocyte contains numerous mitochondria and is attached to adjacent myocytes by intercalated discs and adherens junctions (arrows). Decondensing chromosomes (asterisk) and an incompletely formed nuclear envelope can be observed as well as a peri-nuclear centriole (arrowhead). (**F′**) Higher magnification of (**F**) showing decondensing chromosomes, incomplete nuclear envelope, and the perinuclear centriole. (**F″**) Higher magnification of mitotic nucleus with a zone of reconstructed nuclear envelope and adjacent mitochondria. (**G**) Interaction between an electron-dense transitional cardiac myocyte and an adjacent electron-lucent trabecular myocyte with minimal sarcomeric structure at the junctional region. (**G′**) Higher magnification showing connection of the two cells via desmosomes (arrow), and (**G″**) coupling through an intercalated disk (asterisk). (**H**) Reorganized pattern of the junctional region showing an electron-lucent cardiac myocyte (center) with sparse sarcomeric structures surrounded by and intercalated (arrow) with multiple electron-dense transitional cardiac myocytes (TEM). Scale bars: **A**–**C** = 100 µm, **D** = 1 µm, **E** = 10 µm, **F**–**G** = 2 µm, **H** = 1 µm.

We further characterized the cauterized ventricle in 2 μm thick toluidine blue-stained sections and by transmission electron microscopy (TEM). Observations in plastic sections were consistent with the gross ventricular muscle damage observed in brightfield and SEM, providing additional structural details. The uninjured heart displayed the expected organizational pattern of an outer compact and inner trabeculated myocardium (Figure 3A). Following injury, toluidine blue-stained sections showed three distinct regions: the apical injured area, the remote structurally undisturbed area, and a narrow border region intercalated between them (Figure 3B). Observations in these sections were consistent with the gross ventricular muscle damage observed in brightfield and SEM. The injured apical region revealed a complete loss of myocardial structures. However, by 14 days, trabecular myocytes projected into the apical ventricular lumen and the reconstituted compact heart contained numerous vessels profiles (Figure 3C). Observations by TEM showed that in the cauterized heart at 14 dpc, regions distal to the injury comprised cardiac myocytes (CMs) containing well-defined sarcomeres densely filled with actin–myosin filaments and well-organized z-bands (Figure 3D). By contrast, the repairing apical region of the ventricular myocardium was characterized by the presence of cardiac myocytes with differing levels of structural maturity (Figure 3E). At the lateral edges of the repairing region, transitional cardiac myocytes (TCMs) that included a subpopulation of electron-dense transitional cardiac myocytes (EDTCMs) could be readily identified in the junctional region between the compact myocardium and the spongy heart, reflecting the general structure of the uninjured heart. However, at that time point, the compact myocardium appeared much less organized compared to that of the non-injured heart. In addition, the reconstituting spongy heart contained cardiac myocytes with a marked paucity of sarcomeres, their structural characteristic ranging from nascent to structurally immature, or less fully differentiated (Figure 3E′,E″). Among the structurally immature myocytes, trabecular CMs with mitotic characteristics such as chromosomal structures or incomplete nuclear membrane could be observed (Figure 3F,F′,F″).

In contrast to the lateral edge of the injury at 14 dpc, the junctional region of the medial apical area appears absent or was poorly defined, or the transitional cells were inappropriately localized. As a result, the separation of the compact and spongy heart was less discernible. In that region and, for the most part, cardiac myocytes were separated or displayed loose apposition to each other. Where present, electron-dense and filament-rich EDTCMs were connected to adjacent structurally immature CMs (Figure 3G). Desmosomes and adherens junctions could be seen linking these phenotypically heterogeneous CMs (Figure 3G′,G″). In some instances, multiple EDTCMs could be seen bordering structurally immature electron-lucent CMs, illustrating a level of disorganization of the junctional region (Figure 3H).

### 3.4. Inflammation and Remodeling in the Cauterized Zebrafish Ventricle

**Figure 4:**
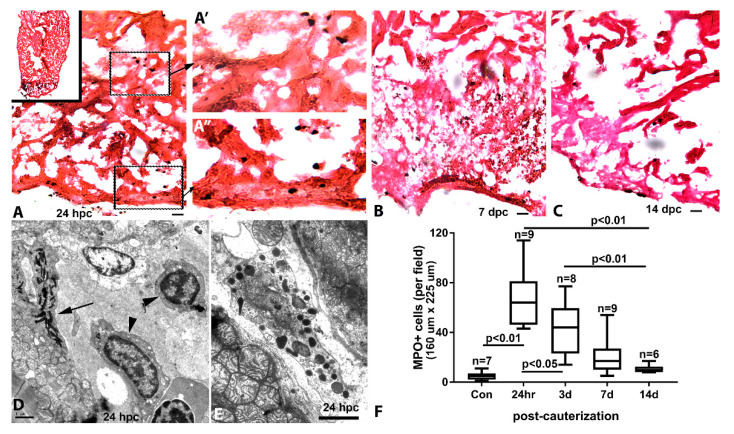
Inflammation in the cauterized zebrafish heart. (**A**) Myeloperoxidase (MPO)-reactive inflammatory cells (black) in a 24 h post-cautery (hpc) ventricle counterstained with eosin (orange). MPO reactivity is localized primarily in the injured region (inset). MPO-positive inflammatory cells in the cauterized apex shown at higher magnification in the spongy myocardium (**A′**) and the compact and junctional region of the myocardium (**A″**). MPO-reactive cells in the necrotic apical region at (**B**) 7 days post-cautery (dpc) and (**C**) 14 dpc. (**D**) Transmission electron micrograph of inflammatory cells (arrowhead) in the lumen of the injury border zone and a heterophil attached to an adjacent cardiac myocyte (arrow). (**E**) A granule-filled inflammatory cell between two adjacent injured cardiac myocytes at 24 hpc. (**F**) Quantitation of MPO-positive cell infiltrate in the injured area over a 2-week period post-cauterization. Scale bars: **A**–**C** = 10 µm, **D**–**E** = 1 µm.

We studied the inflammatory response in the cauterized heart using myeloperoxidase (MPO) reactivity. The uninjured zebrafish ventricle contained few luminal MPO-positive cells and displayed no other histological indication of inflammation. At 24 hpc, the injured apical zone (Figure 4A) contained significant accumulation of MPO-positive cells. These cells could be found distributed throughout the amorphous necrotic region, the injury border zone (Figure 4A′), and in the injured compact heart (Figure 4A″). At 7 dpc, MPO-positive cells remained markedly elevated in the injury area. While some MPO-positive cells could be found in the compact heart of the remote region, inflammation was primarily restricted to the injured area (Figure 4B). By 14 dpc, concomitant with reduction in the size of the injured area, the number of MPO-positive cells was significantly decreased (Figure 4C). Further observations of the cauterized ventricle by TEM revealed a heterogeneous population of inflammatory cells including heterophils, macrophages (Figure 4D), and granulocytes (Figure 4E). From a peak in inflammation at 24 hpc, the inflammatory infiltration markedly subsided after two weeks (Figure 4F).

**Figure 5:**
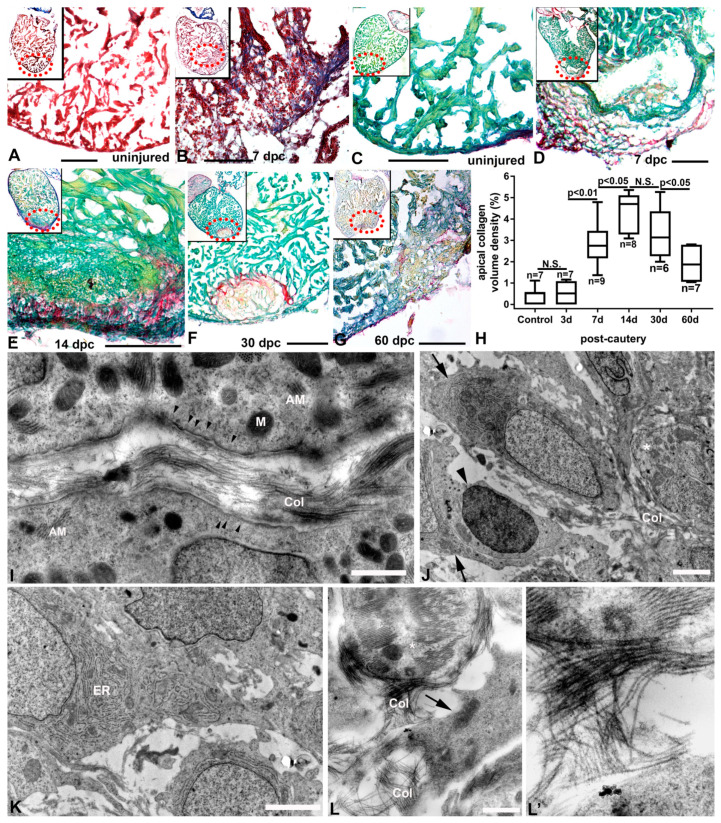
Collagen accumulation in the cauterized zebrafish ventricle. (**A**) Trichrome-stained section of an uninjured ventricle showing little collagen. (**B**) Section of the apical region of a heart at 7 days post-cautery (dpc) with marked accumulation of collagen (blue) in the injured and border zone region. Biebrich Scarlet stained the necrotic region (lower region on the panel) and truncated trabecular myocytes (upper part of the panel); inset shows the extent of the injury and the regional collagen accumulation. (**C**–**G**) Fast Green- and Sirius Red-stained sections in uninjured and cauterized hearts, showing accumulation and resorption of collagen (red) interspersed with remodeled cellular material (green) in the previously necrotic region. Magnified areas shown are from areas within dashed ellipses in upper left insets. (**H**) Quantification of collagen from control to 60 days post-cautery injury. (**I**) TEM of the injury border zone at 14 dpc, showing thick bundles of filamentous collagen fibers (Col) between trabecular myocytes of the compact and spongy heart (AM, actin–myosin bundles; M, mitochondrion; arrowhead, caveolae). (**J**) TEM of an injured and poorly organized compact (cortical) myocardium of a 14 dpc heart, showing loosely associated low sarcomere-containing cardiac myocyte (asterisk) and fibroblasts (arrows). An inflammatory cell (arrowhead) could be seen attached to a fibroblast. (**K**) TEM of activated fibroblasts in the same region showing extensive rough endoplasmic reticulum (ER) and collagen accumulation in the interstitial space. (**L**) TEM of the junctional region at 14 dpc with a thick bundle of collagen decorating the basal aspect of a trabeculated myocyte (asterisk) and a fibroblast (arrow). (**L′**) Higher magnification micrograph showing a bundle of collagen in contact with the cardiac myocyte and the fibroblast. Scale bars: **A**–**G** = 100 µm, **I**–**L** = 1 µm.

Collagen accumulation is an important component of cardiac remodeling. We studied the extent and distribution of collagen during ventricular repair using Masson’s trichrome. Little collagen was observed in the uninjured ventricle (Figure 5A). However, collagen accumulation was pronounced in the injured area from the compact heart to the border zone by day seven post injury (Figure 5B, blue staining). Collagen accumulation was also studied using Fast Green and Sirius Red staining, and little collagen was detected in the uninjured ventricle (Figure 5C). Following injury, significant collagen deposition was seen at 7 dpc (Figure 5D), and at 14 dpc (Figure 5E) when cardiac myocytes were repopulating the injured area. Collagen levels were still elevated at 30 dpc (Figure 5F) but began to decrease at 60 dpc (Figure 5G,H). Transmission electron microscopy studies confirmed the presence of thick bundles of filamentous collagen fibers interspersed between structurally less differentiated and sarcomere-poor cardiac myocytes of the regenerating trabeculae (Figure 5I). The collagen bundles could also be found apposed to the basement membranes of cardiac myocytes. In the compact myocardium, extensive collagen fibers were seen mixed with amorphous materials containing inflammatory cells and numerous activated fibroblasts in close proximity and adjacent to cardiac myocytes (Figure 5J). In particular, the activated fibroblasts contained extensive networks of rough endoplasmic reticulum (Figure 5K). In the junctional region, the proximal ends of trabeculae were decorated with abundant collagen bundles on the basal aspect of cardiac myocyte membranes (Figure 5L). These collagen bundles connected cardiac myocytes with interstitial fibroblasts in the junctional space and formed organized meshes containing fibers that ran parallel and perpendicular to the cells (Figure 5L′).

### 3.5. Re-Innervation of the Injured and Regenerating Zebrafish Heart

**Figure 6:**
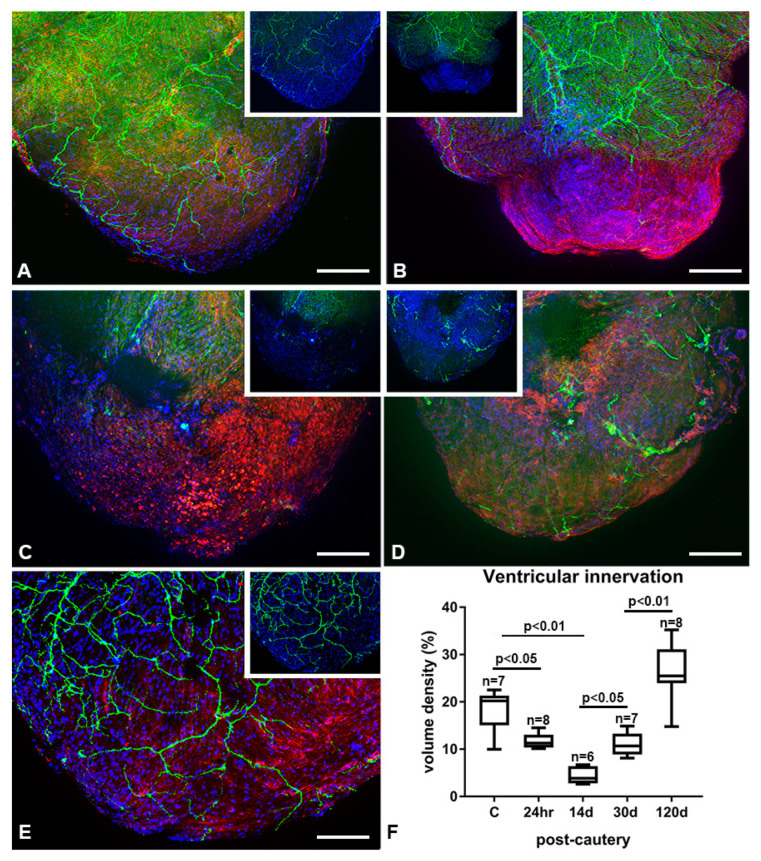
Re-innervation of the regenerating cauterized heart. (**A**) Zn12-stained nerves (green) with wheat germ agglutinin (WGA, red) and Hoechst (blue) in the uninjured ventricle. (**B**) Zn12 immunoreactivity (green) in a ventricle at 24 h post-cautery, showing absence of nerve fibers in the injured apical area. (**C**) Further loss of zn12 staining at 14 days post-cautery (dpc). Zn12 staining at 30 (**D**) and 120 dpc (**E**) in the injured heart. (**F**) Volume density of nerve fibers in the cauterized and regenerating ventricular myocardium. Scale bar = 200 µm.

To determine the effect of apical myocardium cauterization on the ventricular innervation of the zebrafish heart, we studied zn12 immunoreactivity up to 120 dpc. Uninjured adult zebrafish ventricles displayed a relatively parsimonious pattern of nerve fibers (Figure 6A). The zn12-stained nerve network was significantly decreased at 24 hpc (Figure 6B) in the injured area. More specifically, the network was preserved from the base of the ventricle to the border zone of the injury, where it ended abruptly and was absent in the apical injured area. The nerve fiber density was further decreased at 14 dpc with an apparent loss at the border zone (Figure 6C). However, the network of nerves began to progressively return to the injured and regenerated area by 30 dpc (Figure 6D). Re-innervation to pre-injury levels was not achieved until 120 dpc (Figure 6E,F).

## 4. Discussion

In this study, we demonstrate that cauterization is an effective and reliable method to create thermal necrotic injury in the adult zebrafish heart, enabling studies of cardiac remodeling and regeneration. Various approaches to ventricular injury have been used to model myocardial infarction. Apical amputation of the ventricle and cryoinjury are the most often used methods to initiate a regenerative response in the zebrafish [20]. Cryoinjury was developed to study cardiac regeneration in the presence of necrotic tissue [36,37,38]. While each of these approaches present their particular advantages and limitations, their results continue to demonstrate the robust regenerative capacities of the zebrafish myocardium. Our lab has previously used cauterization as an alternative method to create ventricular necrotic regions in the giant danio and goldfish heart [39,40]. We have extended the use of this particular approach to study the injury response in the zebrafish ventricle in the presence of voluminous necrotic tissue. Our results are consistent with previous findings; in particular, they closely parallel the repair and regenerative progression documented in the response to cryoinjury. The sequence of well-defined and temporally overlapping processes of necrosis, inflammation, tissue remodeling, regeneration, and the re-innervation of regenerated tissue support the use of apical cardiac cauterization in the study of the regenerative response in the zebrafish heart.

In these studies, the contact-mediated thermal ablation of myocardial structures by the heated nichrome probe was documented through a variety of means, including the observation of the generated clot under brightfield, identification of tissue necrosis in toluidine blue-stained sections, and confirmation of tissue damage and loss of trabecular structures by SEM and TEM. These approaches were also used to document repair and regeneration of the cauterized ventricle. In the limited number of fish species studied thus far, cardiac regenerative ability appears to range from modest to robust. Regeneration appears to be absent in the medaka [41] and the cave-dwelling *Astyanax mexicanus* [42], similar to the lack of regeneration observed in adult mammalian hearts. The molecular mechanisms underlying the differential responses in these various fish species are not fully known, but are beginning to be elucidated [43]. However, in all cases, cardiac remodeling of various degrees is reported. Here, we demonstrate that cautery injury of the zebrafish ventricle produces an injury response similar to that seen in cryoinjury, including short- to medium-term inflammation (prominent in the first two weeks) and long-term deposition of collagen (weeks to months), coupled with angiogenesis and regeneration. The interplay between remodeling, scarring, angiogenesis, and regeneration in mammalian hearts remains a subject of considerable interest.

A preponderance of evidence supports the idea that in zebrafish, regenerated cardiac tissue relies upon a population of proliferating resident ventricular cardiac myocytes [44,45]. Studies also suggest that regeneration may be driven by cardiac ventricular myocytes of various clonal origins [32,46], endogenous reprogramming [35,47], and permissive ploidization [48]. Similar to indices of regeneration seen in the giant danio heart [39], the regenerating cauterized myocardium of the zebrafish contains myocytes showing a range of structural maturation by two weeks post-cauterization. At that time, a majority of myocytes in the regenerating muscle was noted to have sparse actin–myosin filament and structurally immature sarcomeric structures. However, desmosomes and adherens junctions could be readily observed, indicating electromechanical coupling. The presence of these structurally immature cardiac myocytes further supports the notion that partial structural dedifferentiation accompanies myocardial regeneration in the adult zebrafish ventricle. The extent of dedifferentiation necessary for cardiac myocyte proliferation is not known.

We have previously described the ultrastructural characteristics of the junctional region between the compact (cortical) and spongy trabeculated myocardium in the uninjured zebrafish heart, documenting the structural heterogeneity of cardiac myocytes in that region [49]. In particular, we identified a set of relatively elongated and flattened cardiac myocytes, the transitional cardiac cells (also called primordial), that integrate the compact and spongy heart. The location of the transitional cardiac myocytes overlaps with that of a subpopulation of careg-expressing cardiac myocytes in the junctional region that has also been identified. These findings further support the phenotypic heterogeneity of myocytes in this region, while also providing evidence of potential molecular differences [50]. Indeed, in the setting of regeneration following cryoinjury, the careg-expressing cells appear to be resistant to proliferation, and hence may contribute little to regeneration. In the setting of cautery injury, our TEM observations have identified areas of the regenerating ventricle where the organization at the junctional region is disrupted. In these areas, the transitional cells are not easily recognized, and the electron-dense transitional cardiac myocytes appear to lack proper alignment. Differences in structural and electrical activation patterns have also been reported in repaired and regenerated hearts [51]. Our results suggest that structural pattern alteration in the junctional region, and the absence, or misalignment, of cardiac transitional cells, may create the substrate for disturbance in synchronized conductivity. While further studies are necessary to test this hypothesis, it is conceivable that the proper rearrangement of the regenerated compact and spongy myocardial cells, as well as the interceding transitional cardiac myocytes, may be required for the proper electromechanical function of the regenerated tissue.

Finally, our study documents the innervation of the cauterized and regenerated zebrafish myocardium. In vertebrates, homeostatic maintenance of cardiovascular function is predicated on the presence of both extrinsic and intrinsic autonomic innervation of the heart chambers [52,53,54]. The cardiac innervation of the adult zebrafish has been described in significant detail [55]. Nerves also play an important role during organ regeneration. Indeed, the regeneration of limbs in amphibian species is nerve dependent [56,57]. Nerves are also required in heart regeneration in both the neonatal mouse and zebrafish [58]. In that study, nerve loss from apical amputation was documented in newly regenerated cardiac tissue 30 days post injury. Our studies demonstrate that from the loss of innervation caused by cardiac cauterization, nerve fibers reinvested the remodeled and regenerated myocardium. However, in contrast to the myocyte replenishment and revascularization evident within 2 weeks of injury, the first observable evidence of re-innervation appeared at 30 dpc, and nerve densities comparable to non-injured hearts were observed by 120 dpc. We conclude that among the overlapping processes supporting the regenerating zebrafish heart, innervation is relatively delayed. This finding suggests that nerve factors supporting regeneration may not come from nerve fibers in the regenerating area but could be released from remote nerve fibers or secreted by cells other than nerves in the regenerating area.

## 5. Conclusions

In summary, we show thermal cauterization of the zebrafish heart serves as a reliable injury model with which to study cardiac regeneration. With cautery injury, the early repair response, cardiac remodeling, and the medium- to long-term regeneration processes temporally replicate the sequence described in the cryoinjury model. However, a major advantage of this model is the ease with which locally targeted and voluminous necrotic tissue can be generated using basic and inexpensive equipment found in most laboratories. This approach adds an additional and useful tool to the technical toolbox available for the zebrafish.

## Supplementary Materials

The following are available online at https://www.mdpi.com/2308-3425/7/4/41/s1, Figure S1: Tools and approach to ventricular cautery injury.
