# Supplementary material for: Cauterization as a Simple Method for Regeneration Studies in the Zebrafish Heart
Source: J Cardiovasc Dev Dis. 2020 Oct 3;7(4):41. doi: 10.3390/jcdd7040041 (PMC7711552; doi:10.3390/jcdd7040041)
Supplement: Supplementary file 1 [file jcdd-07-00041-s001.pdf]

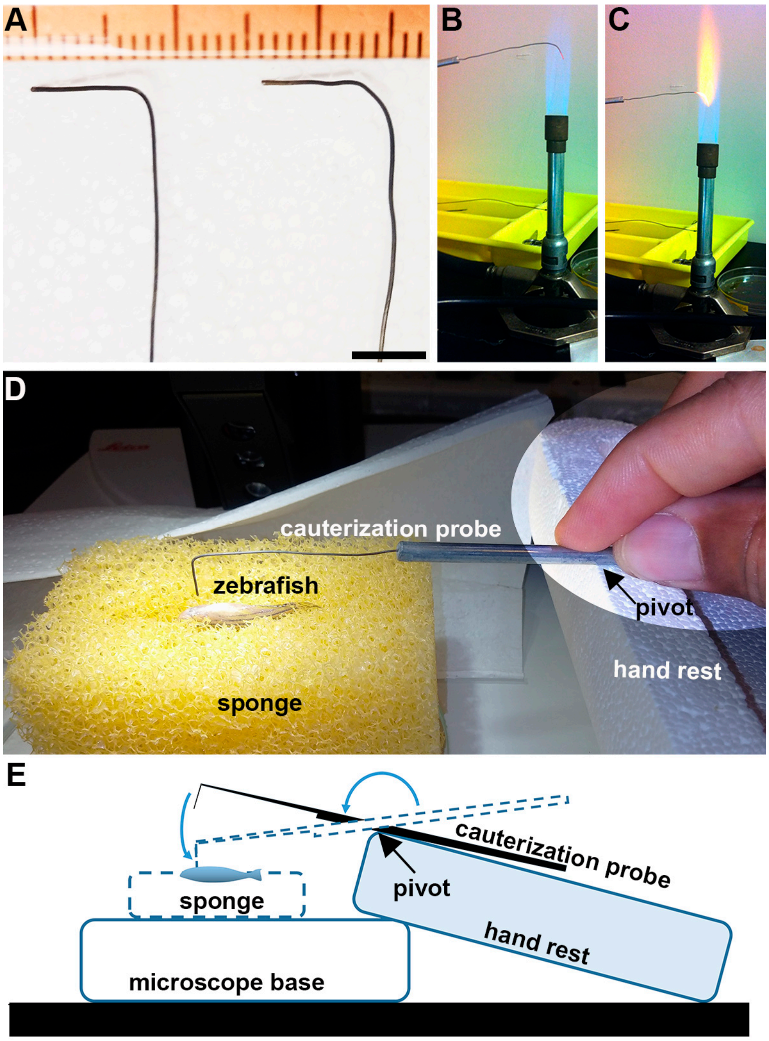

Figure S1. Tools and Approach to Zebrafish Ventricular Cautery Injury. A) Multiple Nichrome inoculating needles may be bent distally at approximately 90 degrees to be used as the cauterization probe; the tips of the probes are sanded to smoothen sharp edges (scale bar = 5 mm). B) The distal tip of a probe is introduced to the flame of a Bunsen burner. C) The tip of the heated probe after 4 seconds. D) A sponge block with a 4 to 5-inch long slit (10 to 13 cm), and approximately half an inch deep (1.3 cm), is used to cradle the anesthetized zebrafish (forceps and exposed ventricle not shown). The sponge block sits on the dissecting microscope base. A rectangular piece of Styrofoam (the top cover of a Styrofoam box) is leaned at the edge of the microscope base to serve as a hand rest and a fulcrum for the probe. Fingers (highlighted) are used to manipulate the probe. E) Schematic diagram of the setup including the sponge block and the anesthetized zebrafish. The probe is made to rest the Styrofoam hand rest. The upper edge of the hand rest also serving as a fulcrum for the probe. This arrangement allowed the probe to be rotated around the pivot and target the apical aspect of the zebrafish ventricle.
